# Trapping *Callinectes sapidus* (Atlantic Blue Crab) in the Mediterranean: What Can Be Learned from *Carcinus maenas* (European Green Crab) Pest Management

**DOI:** 10.3390/ani16101488

**Published:** 2026-05-12

**Authors:** Jonathan W. Burnett, Hannah Ohnstad, Jorg D. Hardege, Helga D. Bartels-Hardege

**Affiliations:** School of Environmental and Life Sciences, Biological Science, The University of Hull, Hull HU6 7RX, UKh.hardege@hull.ac.uk (H.D.B.-H.)

**Keywords:** blue crab, green shore crab, management, tools, control methods, olfactory cues

## Abstract

Invasive marine crustaceans present ecological and socio-economic challenges, particularly where high fecundity, behavioural plasticity, and environmental tolerance limit the effectiveness of conventional control measures. The Atlantic blue crab, *Callinectes sapidus*, has expanded across the Mediterranean, where it exerts strong predatory pressure on native species and aquaculture resources, yet management efforts rely largely on traditional baited trapping. Drawing on extensive evidence from the global management of the European green crab, *Carcinus maenas*, we evaluate why conventional trapping has failed to achieve sustained population suppression in invasive brachyuran crabs. We synthesise lessons from green crab control to identify common biological and operational constraints, including rapid compensatory population responses, bycatch, and limited behavioural selectivity. We then examine the potential of semiochemical-based strategies—incorporating olfactory cues—within an integrated pest management (IPM) framework. Semiochemical-enhanced approaches offer the capacity to directly exploit species-specific sensory ecology, improve trap selectivity, and reduce non-target impacts, addressing fundamental limitations of existing methods. We argue that prioritising such IPM, rather than further optimisation of traditional trapping alone, represents the most realistic pathway for long-term mitigation of *C. sapidus* in the Mediterranean. While eradication remains improbable, strategically deployed semiochemical-based control could substantially enhance suppression efficiency and inform broader invasive species management.

## Simple Summary

Invasive species such as the Atlantic blue crab, *Callinectes sapidus*, cause major disruptions of ecosystems that often lead to declines in native species, change ecosystem function and negatively impact local fisheries and communities (i.e., by wiping out entire mussel populations). Monitoring invasive species and developing mitigation or eradication scenarios is vital, but we often lack the tools to undertake these tasks. In marine crustaceans, trapping is usually the selected method; however, it relies heavily on using non-discriminative fish baits, which also negatively impacts native species and incurs significant environmental and monetary costs. To develop affordable, sustainable management strategies, we evaluate the management of a different invasive crab, the European green crab, *Carciuns maenas*, to learn lessons gained over the past decades. We conclude that integrated pest management requires investment into the development of new techniques that are based on natural olfactory cues. These odours influence and control all crabs’ behaviour including foraging and anti-predator responses, are environmentally friendly natural products, and usually do not incur problems with habituation. Only once such techniques become available at reasonable costs can effective monitoring and selective, regional eradication or control strategies be sustained.

## 1. Introduction

### Callinectes Sapidus, as Invasive Species in the Mediterranean

*Callinectes sapidus*, the Atlantic blue crab, is an intertidal decapod native to the western Atlantic Ocean that has recently emerged as a significant invasive species in the Mediterranean Sea [1]. First detected in 1948 [1], though found as early as 1935 in the north Aegean Sea [2], the species is thought to have been introduced through ballast water discharge and has expanded its presence notably in recent decades, with substantial populations now observed in areas including the Adriatic Sea, and both the eastern and western Mediterranean [1]. This euryhaline species inhabits estuaries, lagoons, and coastal habitats, thriving in a variety of salinities and temperatures [3]. High adaptability and reproductive capacity are attributes enabling *C. sapidus*’ invasive success. It is also an opportunistic feeder with an aggressive nature, enabling it to outcompete native species for resources, leading to significant alterations in local biodiversity and trophic dynamics [1]. According to Regulation (EU) No 1143/2014 of the European Parliament and of the Council of 22 October 2014 on the prevention and management of the introduction and spread of invasive alien species, the blue crab is now designated as an invasive species with a high risk of biodiversity loss. Measures should be undertaken towards control of its population outbreaks either through eradication or minimisation of its population size (Regulation 1143/2014).

*Callinectes sapidus*’ establishment in the Mediterranean poses both ecological and economic challenges as highlighted in Table 1. Ecologically, the species disrupts pre-established food webs, preying on a broad range of native organisms, including fish and shellfish (some of which are commercially important), and drives declines in native populations [3]. From an economic standpoint, the predation on valuable fishery resources combined with *C. sapidus*’ ability to outcompete native species has led to substantial financial losses to local fisheries and aquaculture [1]. For example, Manila clam production plummeted by almost 100% in Sacca del Canarin, with 75% losses in Sacca degli Scardovari (Po Estuary, Adriatic Sea). This equates to an approximate 1000 tonnes of clams in just a 3-month period for these two locations alone, which, according to local producers, is an annual financial loss exceeding €250 million [4]. This figure does not include damages to fishing gear and infrastructure as a direct result of the species’ burrowing behaviour, which further exacerbates these losses [3], as well as the expense of foreign importing of clam seeds from the USA and Portugal to recover stocks [5].

There is a growing interest in establishing a *C. sapidus* fishery in the Mediterranean as a food resource. The species is noted for its high nutritional value, with meat rich in protein, omega-3 fatty acids, vitamins, and minerals [6], and whilst utilising *C. Sapidus* for human consumption could potentially mitigate some of the negative impacts of their invasiveness (i.e., creating new economic opportunities via the development of such fisheries [7]), there are environmental concerns regarding waste from crab processing, particularly shells. However, due to crustacean shells being a source of valuable compounds such as chitin (used in a variety of products including eco-friendly alternatives to plastic [8,9]), there are efforts being made to promote end uses for this resource [3,10].

This review evaluates the limitations of current trapping strategies for *C. sapidus* across its invasive ranges by comparing them to the efforts, challenges and shortcomings in trapping a similarly invasive brachyuran crab, *Carcinus maenas* (the European green crab). Given that current trapping efforts (see Table 2) are not working, combined with rising costs, we aim to critically evaluate if it is sensible to employ the same often flawed strategies used in green crab control, which unsuccessfully aimed to eradicate the invader. Here, we propose that research into alternative, tailored control solutions for monitoring and minimising the impacts of invasive crustaceans is urgently required.

The aim of this review is not to catalogue the impacts of *C. sapidus* in the Mediterranean, but to critically assess why current control strategies have reached an effectiveness ceiling and how management can be redesigned using biologically informed tools. By explicitly comparing blue crab invasion dynamics with the well-documented global management experience of *C. maenas*, we identify recurring limitations of conventional trapping and argue that these failures are rooted in crab sensory ecology and behaviour rather than trap design alone. We propose that semiochemical-based interventions, embedded within an integrated pest management (IPM) framework, offer a mechanistic and scalable route to improving control outcomes. In doing so, this review provides a management-oriented synthesis intended to guide future research investment and operational decision-making for invasive marine crustaceans in the Mediterranean and beyond. Generative artificial intelligence (ChatGPT [Model: GPT-4o, 2026] OpenAI, San Francisco, CA, USA) was used exclusively to assist in formatting and generating table layouts/graphics using data compiled during literature review by the authors. All AI-generated outputs were manually reviewed and verified for accuracy by the authors prior to inclusion in the manuscript. All analysis and interpretation of data/information were conducted by the authors.

**Table 1:** Ecological and economic impacts of *Callinectes sapidus*.

Region	Impact Category	Type	Consequence	Cost (Estimated/Proxy)	Reference
Adriatic (Po Delta)	Economic	Clam predation	Collapse of Manila clam fishery	€65 million/year	[4]
Adriatic (Po Delta)	Economic	Fishery disruption	Small-scale fishery losses	€0.18 million/year	[4]
Adriatic (Po Delta)	Socio-economic	Gear damage	Higher costs, reduced income	~30% income reduction	[11]
Adriatic (Po Delta)	Ecological	Predation	Decline in bivalves, fish, crabs	Not quantified	[12]
Iberian Atlantic	Ecological	Predation	Decline in native molluscs and crustaceans	Not quantified	[13]
Iberian Atlantic	Economic	Fishing gear damage & catch loss	Damage to nets and reduced catch	€0.1–0.3 million/year	[13]
Western Mediterranean	Ecological	Competition	Displacement of native crabs, reduction in clams	€0.7 M in 2024	[14]
Western Mediterranean	Economic	Reduced catch of target fish & bivalves	Lowered yield in artisanal fisheries	€0.2–0.5 million/year	[11]
Eastern Mediterranean	Ecological	Predation & habitat alteration	Reduction in bivalves, crustaceans	Not quantified	[13]
Eastern Mediterranean	Economic	Fishing losses	Decreased artisanal fishery yields	€0.15–0.4 million/year	[13]
Black Sea	Ecological	Predation	Decline in native crab and bivalve populations	Not quantified	[12]
Black Sea	Economic	Fishery disruption	Reduced catches in small-scale fisheries	€0.1–0.25 million/year	[12]
US Atlantic	Ecological	Predation	Reduction of bivalves and juvenile fish	Not quantified	[15]
US Atlantic	Economic	Fisheries and gear damage	Losses in local crab and clam fisheries	$0.5–1.0 million/year	[15]

**Table 2:** Current methods of controlling crustacean invasions.

Species (Invasive)	Invaded Range (Examples)	How Controlled (Current Methods)	Success (Summary)	Notes/Constraints	References
*Carcinus maenas* (European green crab)	Pacific US/Canada (WA, BC, NB), parts of Australia; global introduced range	Intensive removal; targeted commercial/paid harvest pilots; monitoring & rapid response; biosecurity to prevent spread	Partial control (local suppression); eradication generally not feasible once widespread	High effort needed; risk of spread via gear/boats; programmes focus on sustained removal + prevention	[16,17,18,19]
*Callinectes sapidus* (Atlantic blue crab)	Mediterranean lagoons (e.g., Adriatic/Italy), Iberian waters; other non-native coasts	Targeted trapping/harvest; market development/valorisation to incentivise removals; fishery gear adaptation	Partial control (local reduction where harvest sustained); eradication not demonstrated	Economic incentives may help but can risk deliberate spread; management often reactive	[4,20]
*Eriocheir sinensis* (Chinese mitten crab)	European rivers/estuaries (e.g., Thames, Scheldt), parts of North America (e.g., CA)	Trapping during migrations (up/downstream); barriers/screens at intakes; public reporting; regulations on live transport; rapid response planning	Local reduction/containment; eradication rarely achieved	High dispersal and complex life cycle; control focuses on limiting spread and nuisance impacts	[21,22]
*Pacifastacus leniusculus* (signal crayfish)	UK & continental Europe freshwaters	Biocide eradication in small isolated waters; intensive trapping in rivers; containment zoning + licencing	Successful eradication in small isolated ponds; trapping mainly reduces densities (often no eradication)	Biocides non-selective (non-target impacts); eradication feasible only in closed systems	[23,24]
*Procambarus clarkii* (red swamp crayfish)	Southern Europe (rice/agricultural wetlands), Mediterranean catchments; global	Trapping (various designs/baits); habitat & margin restoration to reduce refugia; physical barriers; local harvest	Mostly partial/temporary reductions; eradication generally unsuccessful in open systems	High fecundity and overland dispersal; removal effort must be sustained; reinvasion common	[25,26,27]
*Dikerogammarus villosus* (killer shrimp; amphipod)	UK & Western/Central Europe freshwaters; spread via boats/equipment	Biosecurity/containment (Check–Clean–Dry); decontamination of equipment; feasibility testing of chemical treatments	Containment/prevention emphasised; eradication rarely feasible once established	Small size + high spread risk; chemical treatments constrained by non-target and regulatory limits	[28]
*Hemigrapsus sanguineus* (Asian shore crab)	NW Atlantic (US/Canada); Europe (e.g., Wadden Sea); rocky shores	Physical removal/manual collection; artificial habitat collectors/traps for juveniles; monitoring	Generally unsuccessful at landscape scale; can support local suppression/monitoring	High recruitment; control labour-intensive; best managed via prevention and early	[29]

## 2. Exploring the Experiences from *Carcinus maenas* for *Callinectes sapidus* Management

### 2.1. Why Can We Utilise Comparing Carcinus maenas and Callinectes sapidus?

#### *Carcinus maenas* as Invasive Species

Ranking amongst the top 100 most invasive species in the world (Global Invasive Species Database, 2025), *Carcinus maenas*, commonly known as the European green crab, is a notorious intertidal decapod crustacean native to the northeast Atlantic, ranging from Iceland and Norway to North Africa [30]. Like *Callinectes sapidus*, it is an opportunistic omnivorous predator, consuming a wide variety of prey, including molluscs, crustaceans, and small fish. It is also highly resilient, capable of surviving in low-oxygen conditions [31], exhibiting high tolerance and adaptability to a wide range of fluctuating environmental stressors including pH, salinity and temperature—factors attributed to natural tide cycle exposure in the intertidal zones. This adaptability allows *C. maenas* to thrive in estuarine to fully marine environments [32]. The species’ high reproductive rate allows for rapid population expansion across its native and invasive ranges [31]. Its highly aggressive and agonistic nature, combined with efficient foraging strategies and a lack of natural predators in non-native habitats, all contribute to its success as an invasive species [33], with *C. maenas* often outcompeting native crabs and other benthic organisms for food and habitat [32].

*Carcinus maenas* have established invasive populations on every continent except Antarctica [30], with the North American east and west coasts, South Africa, Australia and parts of Asia all being considered major invasion hotspots [34]. It is thought that ballast water transport, hull fouling, and aquaculture activities were most likely the main mechanisms for this spread, and their expansion along the Atlantic and Pacific coasts has been documented since the early 19th century [30,35]. The severe ecological consequences of *C. maenas* invasions cannot be overstated: from preying upon and decimating commercially valuable shellfish populations, including mussels, clams and oysters [36], to disrupting critical fish and invertebrate habitats such as eelgrass beds [37]. The economic impacts on fisheries and aquaculture industries have resulted in substantial financial losses [33].

### 2.2. Ecological and Behavioural Similarities

Both *C. maenas* and *C. sapidus* exhibit high reproductive rates and opportunistic feeding behaviour with voracious appetites, and their habitats overlap in invasive areas. The twice-annually-reproducing green crabs release around 370,000 young per year [37], whilst the significantly larger, very prolific breeding blue crab females produce 750,000–3,200,000 eggs per spawn with three broods per year, totalling around 9,600,000 eggs [30]. These reproductive capabilities enable both species to establish and expand populations rapidly across their invasive ranges. Both species also share similar diets; they are generalist predators, omnivorous, preying on a variety of bivalves, small fish, and other crustaceans including the cannibalisation of their own species, as well as feeding on algae, eelgrass and other marine plants. This diversity allows the exploitation of a wide range of food sources across ecosystems [15,31], a contributing factor to their ability to outcompete native species and establish dominance. High mobility in both *C. maenas* and *C. sapidus* means both species can traverse long distances, and this is particularly the case during larval dispersal [38,39]. Both species can tolerate fluctuations in salinity, pH and temperature, and this environmental adaptability contributes to their invasive potential [15,37].

There are also some significant differences in the biology and ecology of the two species. These include the use of habitat, with *C. maenas* preferring intertidal zones and rocky substrates and *C. sapidus* being more commonly found in estuarine and soft-bottom habitats [37,39]. Memory and learning capabilities also differ, with Hines [15] noting that *C. sapidus* may have superior associative learning abilities, a factor which may influence or affect interactions with traps and deterrents. Variations in population dynamics are also a consideration. Usually, after initial invasion waves and establishment, *C. maenas* populations are seen to stabilise over time. In contrast, *C. sapidus* populations tend to see fluctuations due to environmental factors such as habitat degradation, as well as overfishing due to the species’ commercial value [31,36].

### 2.3. Learning from Carcinus maenas: Evaluating Trapping Efforts Across Its Invasive Range

#### Trapping Methods Used

Widespread trapping efforts have been employed to control *C. maenas* invasive populations. These methods traditionally include fish-based baited Fukui and box traps [40], such as the model Fukui FT-100 (Fukui North America, Golden Lake, Ontario, Canada), traps that are favoured due to being light-weight, collapsible and durable. Such traps have been implemented for a wide range of crustaceans (Table 2) either for commercial fishing or as control measures for invasive and aquaculture pest species. They can be deployed in large quantities either from shore or boats. These traps feature a rectangular, vinyl-coated high-tensile steel frame covered with square, single-knotted, polyethylene mesh. Two entrances at either end of the trap, where two netting panels form a horizontal “V” with an expandable entry slit at the narrow end, allow crabs to enter [41]. The lure (for example mackerel) is placed in a small bag within the trap. Crabs that approach the trap can enter through open slits featuring a flap mechanism that prevents caught animals from leaving again, making them highly efficient in crab trapping. Crab traps for larger species (such as lobsters that have a legal minimum size for trapping) contain a small escape hole that enables small individuals and bycatch to leave (for design and photos, see [41]).

In some areas, such as the coastal regions of USA and Canada, collapsible minnow traps and hoop nets were deployed, with varying levels of success [42]. This is in part due to the small capacity of modified traps, resulting in rapid trap saturation when deployed in areas experiencing large *C. maenas* populations, thus either deterring crabs from entering the trap (due to being intimidated by the presence of conspecifics) or in some cases allowing captured crabs to escape [43,44]. Other methods saw exclusion fencing and other barriers used to prevent *C. maenas* from entering sensitive habitats such as those in estuarine environments [45]. However, in the case of trapping studies conducted in 2013 at the Haraseeket River in Freeport, Maine (USA), in just two months without maintenance, the structural integrity of fences was compromised [46], highlighting the need for regular upkeep and the labour and costs associated with this method.

### 2.4. Successes, Failures/Complications and Limitations of Trapping Programmes

Whilst trapping programmes have seen widespread implementation, limited long-term success in reducing *C. maenas* numbers has been observed. Parts of the United States and Canada saw some localised successes, with coordinated trapping and monitoring temporarily reducing population numbers enough for short-term ecological recovery, such as seen in the Lummi Sea Pond, though these cases remain exceptions rather than the rule [47]. Due to *C. maenas*’ high reproductive capacity and adaptability, rebounds often occur even in areas with the most intense trapping programmes [48].

A fundamental limitation of conventional crab trapping is the reliance on highly nonspecific attractants, most commonly fish waste or processed fish products, which release broad-spectrum odour plumes that stimulate general foraging responses across multiple taxa rather than species-specific behaviours. Such baits exploit conserved chemosensory pathways associated with scavenging and opportunistic feeding, meaning that traps attract a wide range of non-target organisms including fish, native crabs, lobsters, and other benthic invertebrates [32,37,38]. The basic principle of using decomposing animal matter as bait has remained largely unchanged since antiquity, with functionally similar methods documented in fisheries and trapping practices for millennia, yet without any refinement toward behavioural or taxonomic specificity. As a result, baited traps prioritise odour intensity over selectivity, inherently coupling crab capture with high bycatch rates and necessitating gear modifications such as escape gaps or biodegradable panels that further reduce trapping efficiency [38,47]. This technological stagnation highlights why incremental improvements to trap design alone have failed to resolve bycatch issues and underscores the need for chemically targeted alternatives that exploit species-specific sensory ecology. There are laws in several countries requiring that traps used in crustacean fisheries be equipped with escape holes, biodegradable escape panels or hinges to prevent ghost fishing and allow economically important bycatch to escape [49], overseen by institutions such as NOAA, the National Oceanic and Atmospheric Administration, in the USA. This presents further challenges to efficiency, as many traps fail to capture all size classes of crabs, allowing smaller individuals to escape and continue reproducing/repopulating trapped areas [49].

Another undesirable byproduct of large-scale trapping and removal of a species is unwittingly causing population increases/explosions afterwards; a phenomenon known as the “Hydra effect” [50], named after the Greek myth whereby cutting off the head of a hydra results in two growing back in its place. The phenomenon has been observed in *C. maenas* management in a small estuary in central California (Seadrift Lagoon, Stinson Beach) and occurs because, as a cannibalistic species, *C. maenas* control their own population numbers to a degree, with larger adults preying on juveniles. Due to high reproductive capability (with each female producing over 300,000 eggs per year), with adults removed from the population, the numbers of young go unchecked, with population numbers not only rebounding rapidly but exceeding those prior to trapping [50].

Other factors influencing trapping effectiveness are spatial and temporal variations. Regions experiencing high *C. maenas* abundance in vast coastal regions require intensive trapping to make a discernible impact, making large-scale eradication through trapping virtually impossible. Temporal factors, such as seasonal variations in crab activity, also play a role. As *C. maenas* is more active during warmer months when foraging and reproducing, this makes them more susceptible to trapping [37]. However, colder seasons see vastly reduced movement and migration to deeper water, leading to much lower trapping success. Rising sea temperatures associated with climate change are likely to further complicate these dynamics by extending seasonal activity windows, accelerating growth and reproduction, and increasing metabolic demand in invasive crabs, thereby intensifying both ecological impacts and management challenges [1,15,37]. Warming may enhance trap encounter rates in some regions, but it also risks promoting faster population recovery and range expansion, reducing the long-term effectiveness of removal-based control. Concurrently, changes in seawater chemistry linked to ocean acidification have the potential to alter chemosensory perception, risk assessment, and foraging behaviour in crustaceans, with implications for how crabs respond to both baited traps and chemically mediated cues [51,52]. Together, these stressors highlight the need for management approaches that explicitly account for environmentally driven behavioural plasticity, a challenge revisited in the context of future control strategies below [53].

Resources and funding also represent persistent constraints on the long-term sustainability of invasive crab trapping programmes, with many initiatives on the US west coast and elsewhere relying heavily on short-term funding cycles and community or volunteer labour, which limits both spatial coverage and temporal continuity [38,47]. Empirical data from contemporary west coast management reveals that while intensive trapping can remove substantial numbers of *C. maenas* locally, this does not always translate into sustained suppression at broader spatial scales: for example, coordinated agencies and partners removed nearly 1.7 million green crabs from state waters during winter 2024, yet catch-per-unit-effort and detection rates remain variable across management areas, reflecting backdrop population dynamics rather than consistent control [54]. Focused trapping studies have similarly shown that removal is often effective only in reducing crab numbers temporarily and that changes in environmental conditions or fluctuations in recruitment can quickly reverse short-term declines [19]. In many cases, apparent declines have been followed by rapid population recovery once trapping effort decreases, indicating limited long-term suppression rather than durable control [37,38]. This pattern is supported by recent synthesis showing that contemporary removal programmes, including those employing large-scale trapping on the US west coast, have generally failed to deliver lasting regional suppression of *C. maenas* [48]. Collectively, these findings underline that variability in bait efficacy and trapping methodology alone cannot overcome the biological and operational constraints of conventional removal, reinforcing the need for alternative, behaviourally informed management strategies.

## 3. Developing Management Stratigies

### 3.1. Lessons Learned/Implications for Blue Crab Management Strategies

Trapping efforts both past and present have revealed several key lessons: Traditional control measures that have proven ineffective for *C. maenas*, such as baited trapping, are likely to be equally ineffective in the management of *C. sapidus* [38], due to similarities in behaviour and biology of the two species (Table 2).

Generic approaches result in significant bycatch and limited effectiveness. Replacing traditional baits with more innovative baits may be necessary to improve trapping success [55]. Gear modification and the need for an optimised, targeted, species-specific lure will be critical to success [40]. Additionally, short-term, reactionary responses need to be replaced with continuous long-term, sustained and coordinated monitoring and management, as seen in regions where trapping has mitigated population rebounds [38].

Trapping alone will be insufficient in suppressing invasive populations long-term; integrating trapping with other management techniques (such as the use of semiochemicals including pheromones and kairomones) will be an important consideration. Utilising *C. maenas* commercially has been proposed to incentivise removal, such as GreenCrab.org which released “The Green Crab Cook Book” to promote *C. maenas* as an underutilised sustainable seafood and argues the merits of establishing a green crab fishery in the USA. Similarly, the University of New Hampshire collaborated with Tamworth Distilling to develop “Crab Trapper Whiskey” as an end use for the invader. However, approaches such as these have led to mixed responses, have not to date yielded any results in controlling *C. maenas* numbers, and there are concerns that commercialisation may deter the desire to eradicate [47]. Research into the invasive King Crab (*Paralithodes camtschaticus)* fishery in Norway, where the crab is harvested commercially, revealed that whilst harvesting reduced impacts on the benthos, the net benefits of doing so are uncertain. Analysis indicated that there is currently greater marginal benefit from directing more resources toward baseline research at the frontier of the invasion as opposed to investing in research that focuses on the commercial potential of the invasive crab [56]. Ultimately, while trapping remains a valuable tool in the management of *C. maenas*, it is not a silver bullet. Effective control requires a multifaceted approach that considers ecological dynamics, population resilience, and the socio-economic feasibility of sustained trapping operations (see Table 3) [55].

The fact that *C. sapidus* holds economic value as a commercially harvested species further complicates eradication efforts in non-native regions. Whereas *C. maenas* is predominantly considered a nuisance species across all invasive ranges, *C. sapidus* has established fisheries that may create economic incentives against control measures [15]. The ecological consequences of invasion also differ. Whilst *C. maenas* dominantly disrupts bivalve fisheries and seagrass habitats, the predatory impact of *C. sapidus* extends to local fish populations and native crab species, which may necessitate monitoring on an ecosystem-wide scale as well as adaptive management approaches summarised in Table 3 [39,55]. The implementation of a common management policy and regulations for the invasive blue crab that could be adopted by all EU member countries or ideally even by all Mediterranean countries cannot be taken for granted. Countries exploit the commercial opportunities that blue crabs represent differently and potentially encounter their negative impacts to a different degree. Nevertheless, Mediterranean countries should gain the knowledge of how to control population outbreaks, as significant risks for socioeconomical activities (e.g., fisheries and aquaculture production on a local scale) exist.

### 3.2. Integrated Pest Management and the Role of Semiochemicals

Integrated pest management (IPM) is an ecologically based approach to controlling pest populations. It combines biological, physical, cultural and chemical methods, coordinated to reduce/minimise economic damage and reduce risks to both the environment and human health [62]. Semiochemicals can be broadly defined as chemical compounds that mediate interactions between organisms. They are usually a volatile chemical produced by an organism that incites a response in another [63,64]. They can be categorised as pheromones, facilitating intraspecific communication, such as the ant *Solenopsis invicta* using trail pheromones to recruit nestmates to food sources [65]; as kairomones, which are detected by different species, playing a key role in predator–prey interactions, such as *Daphnia magna* detecting fish kairomones and inducing morphological defences such as spines and helmets as a response [66]; or as allomones, chemicals emitted by an organism to another of a different species for the benefit of the emitting organism, observed in the skunk *Mephitis mephitis*, which releases sulphur-containing compounds to deter predators [67].

The principal advantage of semiochemicals in invasive species management lies in their ability to exploit species-specific and context-dependent behavioural pathways, in contrast to unspecific food-based baits that elicit broadly similar responses across taxa. Chemical cues such as pheromones and kairomones are detected and processed through highly specialised chemosensory systems, allowing responses to be targeted by species, sex, reproductive state, or life history [52,64]. This specificity provides a critical mechanism for reducing bycatch and minimising non-target impacts, a persistent limitation of traditional fish-baited trapping methods that attract a wide range of scavengers and predators [38]. Moreover, semiochemical-based approaches align with ecological sustainability principles by manipulating natural behavioural responses rather than relying on consumptive attractants, thereby reducing bait degradation, habituation, and unintended trophic subsidies [68].

In integrated pest management (IPM) frameworks, chemical cues are used not merely to increase capture rates, but to shape movement, aggregation, and habitat use in predictable ways, enabling strategic deployment of traps and deterrents over space and time [69,70]. These advantages are particularly relevant in aquatic systems, where visual cues are often limited and chemical information dominates behavioural decision-making, making semiochemical-mediated control a more selective and mechanistically robust alternative to unspecific food cues [50,52]. They also present an environmentally friendly alternative to pesticides (that have negative ecological impacts on a broad scale). Miller & Gut [69] noted that semiochemical-based strategies enhance the efficiency of existing management tools whilst also maintaining ecosystem balance via the leveraging of natural behavioural responses. Such management strategies have been applied successfully to several invasive terrestrial species, with great success particularly in controlling insects. Examples include extensively using pheromone traps or lures to manage invaders such as *Pectinophora gossypiella*, the pink bollworm, *Anoplophora glabripennis*, the Asian longhorned beetle, and *Cydia pomonella*, the codling moth [69].

Whilst the use of semiochemicals for controlling invasive aquatic marine species is still very much in its infancy, in part due to the need to identify and purify cues that are effective in aqueous environments, it has proven effective in the Great Lakes, U.S.A., to control freshwater fish. Here, olfactory cues were guiding *Petromyzon marinus*, an invasive sea lamprey, into traps, which have significantly reduced their population numbers [71]. Likewise, research by Stebbing et al. [72] demonstrated that pheromones can be incorporated into population management strategies for *Procambarus clarkii*, illustrating how semiochemicals can enhance the selectivity and behavioural relevance of trapping in invasive freshwater crustaceans.

Similar principles were implicitly recognised in early management work on *C. maenas*, where Behrens, Yamada and colleagues showed that conventional baited trapping alone produced inconsistent and often short-lived reductions in green crab abundance, largely because trap performance was decoupled from crab behaviour, life stage, and sensory ecology [36,47]. These limitations have motivated the development of behaviourally informed trapping technologies, including recent patented approaches that employ non-toxic slow-release matrixes based on natural products such as carboxy-cellulose, chitosan or alginate to deploy defined chemical attractants within controlled-release systems to selectively manipulate crustacean movement and trap entry, rather than relying on nonspecific food baits [73].

As semiochemical-enhanced trapping techniques are targeted, species-specific, and environmentally sustainable, they form a core component of integrated pest management (IPM) rather than a stand-alone control measure [69]. IPM explicitly combines multiple complementary tools—such as chemical attractants, physical capture devices, deterrents, temporal targeting, and monitoring—so that no single method is relied upon in isolation [62]. In terrestrial insect management, for example, pheromone lures are routinely combined with coloured sticky traps, mass-trapping grids, and increasingly with sensor-based monitoring systems to improve detection, reduce non-target impacts, and guide adaptive deployment of control effort [69,70]. This strategic integration is directly transferable to invasive marine crustaceans. In the case of *Callinectes sapidus*, strong reliance on chemical cues for mating, foraging, and habitat selection, combined with high behavioural plasticity, makes the species particularly well suited to IPM frameworks that integrate semiochemical-based attractants with conventional trapping and spatially targeted management, offering a more effective and scalable alternative to reliance on fish-baited traps alone.

### 3.3. Developing a Sustainable Strategy

The global experience of managing invasive brachyuran crabs demonstrates that conventional baited trapping, even when applied intensively, rarely achieves sustained population suppression once an invasion is established. Decades of efforts targeting *C. maenas* have revealed consistent constraints, including high fecundity, rapid compensatory population responses following adult removal, behavioural habituation to traps, and substantial bycatch that limits both efficiency and ecological acceptability [32,38,47,48]. These limitations are not artefacts of inadequate deployment but emerge from fundamental aspects of crab biology. The continued reliance on similar trapping strategies for *C. sapidus* in the Mediterranean therefore risks repeating well-documented management failures rather than addressing their underlying causes.

A key lesson from green crab control is that population suppression cannot be achieved through non-selective removal alone [50]. Trapping strategies that fail to discriminate between sex, reproductive status, or behavioural context are particularly vulnerable to the hydra effect, whereby removal of large individuals relaxes density-dependent constraints and accelerates recruitment [50]. Blue crabs exhibit even greater reproductive output and learning capacity than green crabs [15], suggesting that further optimisation of traditional baits or trap geometry is unlikely to overcome these biological constraints. Consequently, effective management must shift from purely extractive approaches toward interventions that directly manipulate behaviour (Table 3). To optimise the efficiency of IPM, for example, for the application of sex pheromones to trap reproductively active females, detailed knowledge of this critical life history stage of the blue crab and especially the spatiotemporal and bathymetrical distribution of ovigerous females in every invaded coastal area (lagoons, estuaries, bays) within the target areas is essential. Sex-specific targeted olfactory cues have significant potential to drastically minimise the number of reproducing crabs, though adapting trapping for different local populations will be required.

Semiochemical-based strategies provide a mechanistically grounded means of achieving this shift. Both *C. maenas* and *C. sapidus* rely heavily on chemical cues to mediate foraging, mate location, habitat selection, and predator avoidance [51,52]. Exploiting these cues allows management to target specific demographic components of invasive populations, such as reproductively active adults or juveniles occupying nursery habitats. Within an integrated pest management (IPM) framework, semiochemicals can function not only as attractants to enhance trap efficiency but also as deterrents that modify spatial behaviour [70], reduce access to vulnerable habitats, or redirect crabs toward controlled capture zones [70,74]. Importantly, such approaches directly address persistent bycatch issues by increasing behavioural selectivity, a major weakness of conventional trapping [38].

The integration of semiochemicals into IPM should be viewed as a system-level redesign rather than an incremental improvement to existing practices. Behaviourally mediated IPM combines targeted attractants, predator-associated kairomones, strategic spatial deployment, and temporal alignment with key life history events such as mating or juvenile recruitment pulses [62,69]. This framework is particularly well suited to estuarine and lagoonal environments, where invasion impacts are most severe, yet management access is feasible. Evidence from other aquatic invaders, including chemically guided control of sea lamprey (*Petromyzon marinus*) and pheromone-based management of invasive crayfish, demonstrates that semiochemical interventions can be operationally scalable when embedded within coordinated monitoring and adaptive management programmes [71,72,75].

Nevertheless, expectations must remain realistic. As with green crab invasions, eradication of *C. sapidus* from open Mediterranean systems is improbable [47,55]. The objective of semiochemical-enhanced IPM is therefore long-term suppression and impact mitigation rather than elimination. Achieving this will require sustained investment in chemical ecology research, including identification of key cue compounds, optimisation of delivery systems, and evaluation of population-level responses [52,64]. Importantly, the growing economic value of blue crabs complicates management incentives, underscoring the need for governance frameworks that prevent perverse outcomes such as intentional spread or resistance to control [7,15].

Overall, the failure of conventional trapping to control *C. maenas* provides a cautionary precedent for blue crab management in the Mediterranean. Rather than refining methods that have repeatedly underperformed, management strategies should prioritise biologically informed, semiochemical-based IPM approaches that align with crab sensory ecology. Doing so offers the most credible pathway to improving control efficiency while minimising ecological collateral effects and provides a transferable model for managing invasive marine crustaceans more broadly [52,70].

## 4. Conclusions

A review of *C. maenas* trapping efforts has highlighted essential insights into the complexities of managing marine bio invasions, with a key takeaway being the importance of understanding species-specific behaviour/biology when designing measures of control. Traditional trapping methods, including baited traps and net barriers, have shown limited success in achieving population reductions, primarily due to the species’ rapid recolonisation and high fecundity [47]. Additionally, bycatch remains a significant challenge, limiting the specificity and efficiency of these trapping programmes [33]. Despite these challenges, *C. maenas* trapping programmes have provided valuable lessons in invasive species management. The failure to suppress populations highlights the need for more targeted, innovative approaches that go beyond conventional methods.

Given the limitations of traditional trapping methods, semiochemical-based integrated pest management (IPM) offers a promising alternative for controlling *Callinectes sapidus*. Semiochemical lures, including pheromones and predator-associated kairomones, have demonstrated precision and sustainability in enhancing trap performance while minimising bycatch [52]. Unlike traditional baits, which attract a broad range of species, semiochemical lures would selectively target *C. sapidus*, improving trapping efficiency and reducing non-target impacts. Moreover, the use of semiochemicals aligns with ecological sustainability principles. By mimicking natural chemical cues, these strategies minimise disruption to non-invasive fauna and reduce reliance on artificial baits, which often contribute to environmental contamination [64]. Implementing semiochemical-based trapping as part of a broader IPM framework can provide long-term, cost-effective solutions for mitigating the ecological and economic impacts of *C. sapidus* invasions.

To advance the application of semiochemical-based IPM for *C. sapidus*, immediate research efforts should focus on identifying and synthesising key chemical attractants. While preliminary studies suggest that predator odours and mating pheromones should effectively lure *C. sapidus*, further investigation is required to optimise lure formulations and delivery mechanisms. Long-term strategies should prioritise the integration of semiochemical-based trapping within comprehensive IPM frameworks. This includes incorporating molecular monitoring tools such as eDNA analysis to track population dynamics and inform adaptive management approaches [76]. Additionally, mathematical modelling of *C. sapidus* population responses to semiochemical traps can help refine deployment strategies and maximise control efficiency [77].

From a policy perspective, regulatory frameworks should support the adoption of semiochemical-based IPM through funding initiatives, permitting streamlined testing and deployment of novel attractants. Collaborative efforts between researchers, policymakers, and industry stakeholders can facilitate the commercialisation and large-scale implementation of these solutions. The global rise in marine bio invasions underscores the urgent need for innovative, science-driven management strategies. Semiochemical-based IPM represents a paradigm shift in invasive species control, offering a highly targeted, ecologically sound alternative to conventional methods. However, realising its full potential requires sustained investment in chemical ecology research, interdisciplinary collaboration, and policy support for practical implementation. Stakeholders across the scientific, regulatory, and industrial sectors must work together to accelerate the development and deployment of semiochemical-based strategies. Funding agencies should prioritise research on invasive crustacean chemical ecology, while policymakers should establish guidelines for integrating these technologies into existing management frameworks. Public–private partnerships can also drive innovation by fostering collaboration between research institutions and commercial entities specialising in semiochemical lure production.

Finally, it is critical to recognise that eradication is an unrealistic goal for most established marine invasions, including *C. sapidus*. Instead, management efforts should focus on long-term population suppression and impact mitigation. By leveraging species-specific control strategies like semiochemical-based IPM, resource managers can develop more effective, environmentally responsible approaches to invasive species management.

## 5. Patents

Methods of trapping crustaceans. US12317872B2, patent application granted on 3 June 2025, status: active, expiration: 27 April 2042.

## Figures and Tables

**Table 3:** Comparisons of *C. maenas* and *C. sapidus* and management implications.

Trait/Axis	*Carcinus maenas* (European Green Crab)	*Callinectes sapidus* (Atlantic Blue Crab)	Why Comparable/ Management Implication	Key References
Native range & invasion status	Native to NE Atlantic (Europe/N Africa); globally invasive in temperate coasts; established across multiple continents.	Native to W Atlantic; invasive and established across Mediterranean basin and parts of European Atlantic coasts; expanding in recent decade.	Both are eurytopic, highly invasive brachyuran crabs with broad environmental tolerances and strong trophic impacts; rapid spread once established.	[16,57,58]
Habitat breadth (adult)	Intertidal–shallow subtidal; estuaries, bays, lagoons, rocky/muddy substrates; tolerates variable conditions.	Estuaries, lagoons, nearshore marine zones; shallow epibenthic omnivore; uses macroalgal/seagrass nurseries; depth to ~35 m.	Occupy estuary–lagoon mosaics, management access is feasible (trapping, barriers), reinvasion risk is high.	[57,58]
Salinity tolerance	Adults survive broad salinity ranges (~10–50‰ reported); early stages often require higher salinity than adults.	Very broad salinity tolerance reported; metabolic performance suggests tolerance ~0 to high salinities, optimum ~18.5 psu; survives ~5 psu up to hypersaline.	Both are euryhaline with estuary life cycles. Control must target brackish “bottlenecks” (nursery/mating zones) and manage dispersal to adjacent marine larval habitats.	[1,57,58,59,60]
Thermal tolerance & climate linkage	Adults tolerate wide thermal range (~0–33 °C reported); larvae narrower optimum (~10–22.5 °C).	Eurythermal; Mediterranean tolerance ~3–40 °C; optimum ~24 °C (from metabolic studies).	Show strong climate-mediated range potential; warming lengthens active season. Sustained, long-term control required.	[57,58]
Reproductive strategy & fecundity	High fecundity; multiple zoeal stages + megalopa; reproduction often seasonal but flexible with latitude/temperature.	High fecundity; mating in brackish waters; females migrate to higher salinity for larval release; multiple zoeal stages + megalopa typical of Portunids.	Both have high propagule pressure, complex life cycles; management to prioritise removing mature females; or prevent larval export.	[3,56,58,60]
Larval dispersal & connectivity	Planktonic larval phase can disperse widely via currents; human-mediated secondary spread common.	Planktonic larvae develop in marine waters; connectivity between lagoons/coasts facilitates spread; invasion patterns linked to climate and research effort signals.	Local removal can be impacted by larval supply from outside catchment. Coordinate control regionally, pair trapping with early detection.	[16,58,61]
Diet & trophic role	Opportunistic omnivore; strong predation on bivalves and other invertebrates; can restructure benthic communities.	Molluscs, crustaceans, fish, detritus/algae; aggressive/cannibalistic; competes with crabs including *C. maenas*.	Functional similarity (generalist omnivores) supports inference: impacts and control leverage points (prey refuges, habitat complexity) can be analogous.	[16,57,58]
Invasion dynamics (lag–boom)	Often shows lag phase followed by rapid expansion once thresholds crossed (propagule pressure + conditions).	Long latency (~decades) followed by strong expansion from ~2014 onwards.	Both may appear “manageable” early; delays are costly. Early detection + rapid response is the critical transferable lesson.	[16,58,61]
Biotic interactions relevant to limits	Predation can limit populations locally; plan notes predators exist but effects vary; distribution can be shaped by predators (incl. blue crabs).	Predation/competition context-dependent; may prey/compete with other crabs; can act as predator limiting green crab in parts of its invaded range.	Cross-system: strong crab–crab interactions, controlling one invader can release another; management to anticipate community-level responses.	[58,61]
Control evidence base	Best-documented: intensive trapping/removal, early detection, large-scale eradication rarely achievable once widespread; plans emphasise sustained suppression.	Control in invaded Mediterranean largely via trapping/targeted harvest; developing markets; pilot programmes (recreational fishing); evidence for eradication limited.	Transferable lesson: avoid overpromising eradication; invest in sustained suppression + prevention; ensure harvest incentives do not encourage spread.	[11,19,61]

## Data Availability

No new data were created.
